# An ortho­rhom­bic polymorph of *N* ^1^,*N* ^4^-diphenyl-3,6-bis­(phenyl­imino)­cyclo­hexa-1,4-diene-1,4-di­amine

**DOI:** 10.1107/S1600536814006254

**Published:** 2014-03-29

**Authors:** Keiji Ohno, Takashi Fujihara, Akira Nagasawa

**Affiliations:** a Department of Chemistry, Graduate School of Science and Engineering, Saitama University, Shimo-Okubo 255, Sakura-ku, Saitama 338-8570, Japan; b Comprehensive Analysis Center for Science, Saitama University, Shimo-Okubo 255, Sakura-ku, Saitama 338-8570, Japan

## Abstract

A new ortho­rhom­bic polymorph of the title compound, C_30_H_24_N_4_, with a density of 1.315 Mg m^−3^, has been obtained. The mol­ecule is centrosymmetric with the centroid of the cyclo­hexa-1,4-diene ring located on an inversion center. The two unique benzene rings are almost perpendicular to each other [dihedral angle = 86.70 (6)°] and are oriented at dihedral angles of 30.79 (5) and 68.07 (5)° with respect to the central cyclo­hexa­diene ring. In the crystal, π–π stacking is observed between the central cyclo­hexa-1,4-diene-1,4-di­amine unit and a phenyl ring of a neighboring mol­ecule [centroid–centroid distance = 3.7043 (7) Å]. The crystal structure of the triclinic polymorph [Ohno *et al.* (2014 ▶). *Acta Cryst.* E**70**, o303–o304] showed chains running along the *b*-axis direction through weak C—H⋯π inter­actions.

## Related literature   

For general background to the title compound, see: Kimish (1875 ▶). For the triclinic polymorph of the title compound, see: Ohno *et al.* (2014 ▶). For related structures, see: Siri & Braunstein (2000 ▶); Khramov *et al.* (2006 ▶); Boydston *et al.* (2006 ▶); Huang *et al.* (2008 ▶); Su *et al.* (2012 ▶). A calculation using *Gaussian98* indicates that the triclinic form of the title compound is more stable, see: Frisch *et al.* (2001 ▶).
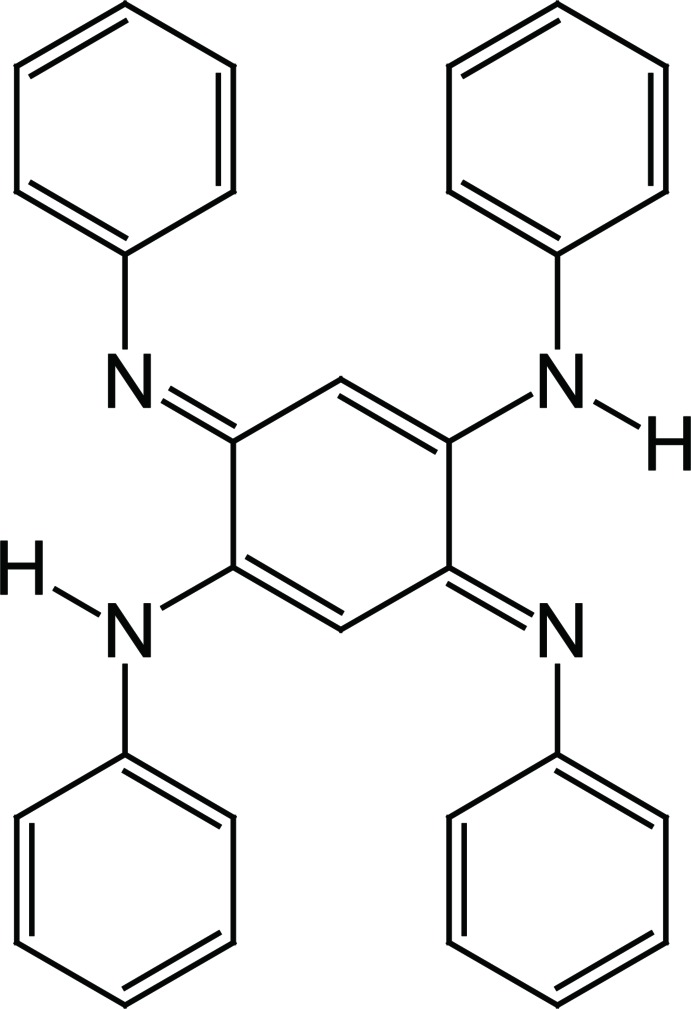


## Experimental   

### 

#### Crystal data  

C_30_H_24_N_4_
*M* *_r_* = 440.53
Orthorhombic, 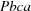

*a* = 9.1927 (5) Å
*b* = 12.4711 (7) Å
*c* = 18.9806 (11) Å
*V* = 2176.0 (2) Å^3^
*Z* = 4
Mo *K*α radiation
μ = 0.08 mm^−1^
*T* = 173 K
0.35 × 0.30 × 0.10 mm

#### Data collection  

Bruker SMART APEX CCD area-detector diffractometer
Absorption correction: multi-scan (*SADABS*; Bruker, 2001 ▶) *T*_min_ = 0.97, *T*_max_ = 0.99
14830 measured reflections
2591 independent reflections
2193 reflections with *I* > 2σ(*I*)
*R*_int_ = 0.116

#### Refinement  

*R*[*F* ^2^ > 2σ(*F* ^2^)] = 0.046
*wR*(*F* ^2^) = 0.133
*S* = 1.09
2591 reflections
158 parameters
H atoms treated by a mixture of independent and constrained refinement
Δρ_max_ = 0.31 e Å^−3^
Δρ_min_ = −0.24 e Å^−3^

### 

Data collection: *SMART* (Bruker, 2007 ▶); cell refinement: *SAINT* (Bruker, 2007 ▶); data reduction: *SAINT*; program(s) used to solve structure: *SHELXTL* (Sheldrick, 2008 ▶); program(s) used to refine structure: *SHELXTL*; molecular graphics: *ORTEP-3 for Windows* (Farrugia, 2012 ▶); software used to prepare material for publication: *SHELXTL*.

## Supplementary Material

Crystal structure: contains datablock(s) global, I. DOI: 10.1107/S1600536814006254/xu5776sup1.cif

Structure factors: contains datablock(s) I. DOI: 10.1107/S1600536814006254/xu5776Isup2.hkl

Supporting information file. DOI: 10.1107/S1600536814006254/xu5776Isup3.cml

CCDC reference: 992862

Additional supporting information:  crystallographic information; 3D view; checkCIF report

## Figures and Tables

**Fig. 1.**
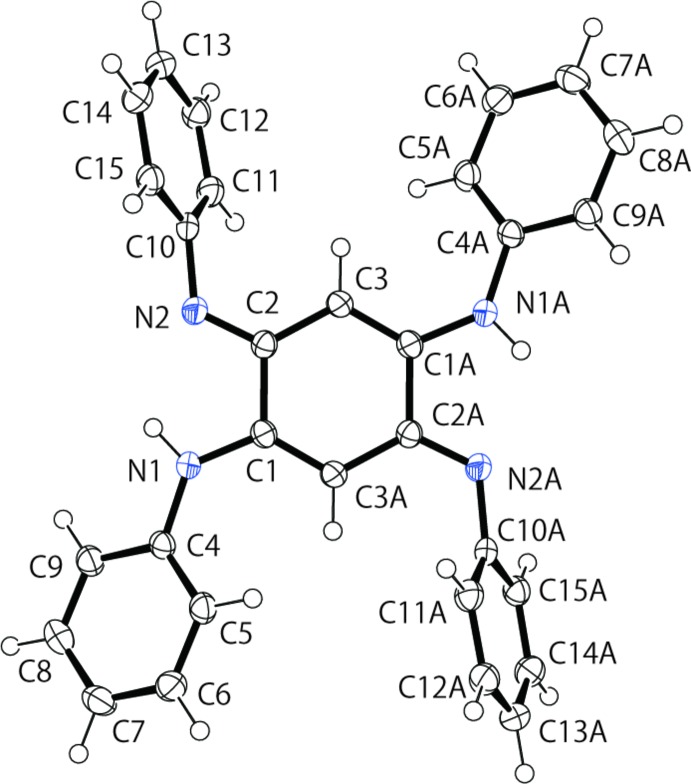
The molecular structure in the orthorhombic polymorph of I, with displacement ellipsoids drawn at the 50% probability level.

**Fig. 2.**
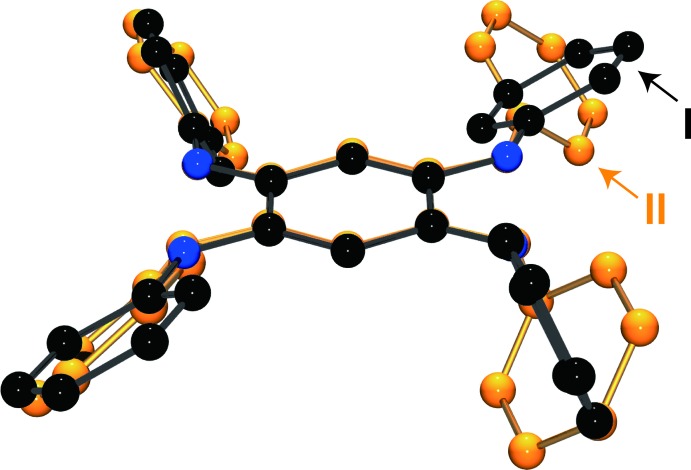
A superposition of the molecular structures of the orthorhombic polymorph of I (colour: black) and the triclinic one (colour: orange).

**Fig. 3.**
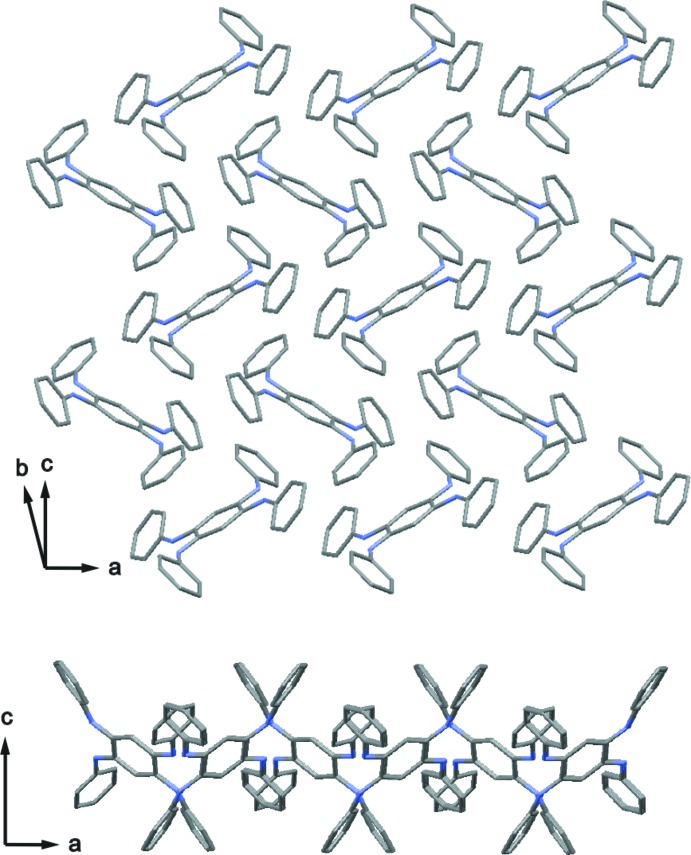
The packing structure through intermolecular π–π interaction in the orthorhombic polymorph of I.

## supplementary crystallographic information

### 1. Comment

*N^1^,N^4^*-Diphenyl-3,6-bis(phenylimino)cyclohexa-1,4-diene-1,4-diamine (I) was synthesized as early as in 1875 (Kimish, 1875) and called azophenine. Recently derivatives of I were prepared and these molecular structures were reported (Siri & Braunstein, 2000; Khramov *et al.*, 2006; Boydston *et al.*, 2006; Huang *et al.*, 2008; Su *et al.*, 2012). Previously, we reported the molecular structure of I in triclinic *P*-1 space group, which is obtained from an oxidation reaction of aniline in the presence of [V^IV^(O)(*η*^2^-ox)(H_2_O)_3_] (ox^2-^ = oxalate) in a mixture of EtOH and H_2_O (Ohno *et al.*, 2014).

We obtained the crystals of I in orthorhombic *Pbca* space group from a reaction with aniline and [V^IV^(O)(*η*^2^-ox)(H_2_O)_3_] and will report here its molecular and crystal structures. This crystal is a porymorph of the previously reported triclinic structure, which showed one-dimensional chains running along the *b*-axis direction through weak C—H···*π* interactions in the crystal.

The crystals contain only I. The main structural difference between the polymorphs of I lies in the orientation of phenyl rings (Figure 2). The neighboring phenyl rings in orthorhombic polymorph of I locate near perpendicular with each other, where the dihedral angle between C(4)—C(5)—C(6)—C(7)—C(8)—C(9) and C(10 A)—C(11 A)—C(12 A)—C(13 A)—C(14 A)—C(15 A) phenyl rings is 86.71°. On the other hand, the dihedral angles between neighboring phenyl rings in triclinic polymorph of I are 29.46 and 19.69° for between C(7)—C(8)—C(9)—C(10)—C(11)—C(12) and C(25)—C(26)—C(27)—C(28)—C(29)—C(30) phenyl rings and C(13)—C(14)—C(15)—C(16)— C(17)—C(18) and C(19)—C(20)—C(21)—C(22)—C(23)—C(24) ones, respectively.

Packing structure of the orthorhombic polymorph of I represented two-dimensional sheets through intermolecular *π*–*π* interaction, where the distances between the phenyl ring C(10)—C(11)—C(12)—C(13)—C(14)—C(15) and the central six-membered ring of adjacent molecule C(1)—C(2)—C(3)—C(1 A)—C(2 A)—C(3 A) is about 3.54 Å (the symmetry code: -*x* + 0.5, *y* - 0.5, *z*) and the dihedral angle between them is 10.70 (8)° (Figure 3).

Calculations using Gaussian98 (Frisch *et al.*, 2001) with a B3LYP/6–31 G(*d*) set of parameters for polymorphs indicate that the triclinic form is more stable than the monoclinic form by approximately 10 kJ mol^-1^.

### 2. Experimental

The V^IV^ complex [V^IV^(O)(*η*^2^-ox)(H_2_O)_3_] was purchased as "VO(ox)^.^*n*H_2_O" from Wako Chemicals, and used without further purification. A solution of aniline (27.9 g, 300 mmol) in EtOH (50 cm^3^) was added to a solution of VO(ox)^.^*n*H_2_O (1.13 g, 3.00 mmol) in a mixture of EtOH (50 cm^3^) and H_2_O (100 cm^3^). The reaction mixture was set aside for 2 weeks at room temperature in air. The precipitated crystals of I were filtered off, washed with H_2_O and EtOH, successively, and dried. Yield 1.34 g. (5.1%). ^1^H NMR / CDCl_3_: *δ* 8.22 (s, 2H, N*H*), 7.41–6.88 (m, 20H, Ph*H*), 6.21 (s, 2H, C*H*). MALDI TOF MS: 441 (*M*+1). UV-vis / CH_2_Cl_2_, *λ*/nm (*ε*/*M*^-1^cm^-1^): 290 (46000), 379 (30000).

### 3. Refinement

The H atoms of NH moiety was located from a Fourier difference map and refined isotropically. Other H atoms were placed at idealized positions with C—H = 0.95 Å, and refined in riding mode with *U*_eq_(H) = 1.2*U*_iso_(C).

### Crystal data

**Table:**

C_30_H_24_N_4_	*F*(000) = 928
*M**_r_* = 440.53	*D*_x_ = 1.345 Mg m^−^^3^
Orthorhombic, *P**b**c**a*	Mo *K*α radiation, λ = 0.71073 Å
Hall symbol: -P 2ac 2ab	Cell parameters from 6200 reflections
*a* = 9.1927 (5) Å	θ = 3.0–27.8°
*b* = 12.4711 (7) Å	µ = 0.08 mm^−^^1^
*c* = 18.9806 (11) Å	*T* = 173 K
*V* = 2176.0 (2) Å^3^	Plate, orange
*Z* = 4	0.35 × 0.30 × 0.10 mm

### Data collection

**Table:**

Bruker SMART APEX CCD area-detector diffractometer	2591 independent reflections
Radiation source: fine-focus sealed tube	2193 reflections with *I* > 2σ(*I*)
Graphite monochromator	*R*_int_ = 0.116
Detector resolution: 8.366 pixels mm^-1^	θ_max_ = 27.9°, θ_min_ = 2.2°
φ and ω scans	*h* = −12→11
Absorption correction: multi-scan (*SADABS*; Bruker, 2001)	*k* = −16→15
*T*_min_ = 0.97, *T*_max_ = 0.99	*l* = −16→24
14830 measured reflections	

### Refinement

**Table:**

Refinement on *F*^2^	Primary atom site location: structure-invariant direct methods
Least-squares matrix: full	Secondary atom site location: difference Fourier map
*R*[*F*^2^ > 2σ(*F*^2^)] = 0.046	Hydrogen site location: inferred from neighbouring sites
*wR*(*F*^2^) = 0.133	H atoms treated by a mixture of independent and constrained refinement
*S* = 1.09	*w* = 1/[σ^2^(*F*_o_^2^) + (0.073*P*)^2^ + 0.1882*P*] where *P* = (*F*_o_^2^ + 2*F*_c_^2^)/3
2591 reflections	(Δ/σ)_max_ < 0.001
158 parameters	Δρ_max_ = 0.31 e Å^−^^3^
0 restraints	Δρ_min_ = −0.24 e Å^−^^3^

### Special details

**Table:**

Geometry. All e.s.d.'s (except the e.s.d. in the dihedral angle between two l.s. planes) are estimated using the full covariance matrix. The cell e.s.d.'s are taken into account individually in the estimation of e.s.d.'s in distances, angles and torsion angles; correlations between e.s.d.'s in cell parameters are only used when they are defined by crystal symmetry. An approximate (isotropic) treatment of cell e.s.d.'s is used for estimating e.s.d.'s involving l.s. planes.Least-squares planes (*x*,*y*,*z* in crystal coordinates) and deviations from them (* indicates atom used to define plane)- 4.5932 (0.0050) *x* + 6.9089 (0.0064) *y* + 12.6394 (0.0086) *z* = 6.3197 (0.0043)* 0.0005 (0.0006) C1 * -0.0005 (0.0006) C2 * 0.0005 (0.0006) C3 * -0.0005 (0.0006) C1_$1 * 0.0005 (0.0006) C2_$1 * -0.0005 (0.0006) C3_$1Rms deviation of fitted atoms = 0.0005- 5.6034 (0.0038) *x* + 5.0416 (0.0058) *y* + 12.9434 (0.0070) *z* = 2.9266 (0.0041)Angle to previous plane (with approximate e.s.d.) = 10.70 (0.08)* 0.0006 (0.0008) C10_$2 * -0.0066 (0.0008) C11_$2 * 0.0067 (0.0009) C12_$2 * -0.0008 (0.0009) C13_$2 * -0.0053 (0.0009) C14_$2 * 0.0053 (0.0008) C15_$2Rms deviation of fitted atoms = 0.0049- 4.5932 (0.0050) *x* + 6.9089 (0.0064) *y* + 12.6394 (0.0086) *z* = 6.3197 (0.0043)Angle to previous plane (with approximate e.s.d.) = 10.70 (0.08)* 0.0005 (0.0006) C1 * -0.0005 (0.0006) C2 * 0.0005 (0.0006) C3 * -0.0005 (0.0006) C1_$1 * 0.0005 (0.0006) C2_$1 * -0.0005 (0.0006) C3_$1 - 3.7228 (0.0017) C10_$2 - 3.4796 (0.0019) C11_$2 - 3.2849 (0.0018) C12_$2Rms deviation of fitted atoms = 0.0005- 4.5932 (0.0050) *x* + 6.9089 (0.0064) *y* + 12.6394 (0.0086) *z* = 6.3197 (0.0043)Angle to previous plane (with approximate e.s.d.) = 0.00 (0.10)* 0.0005 (0.0006) C1 * -0.0005 (0.0006) C2 * 0.0005 (0.0006) C3 * -0.0005 (0.0006) C1_$1 * 0.0005 (0.0006) C2_$1 * -0.0005 (0.0006) C3_$1 - 3.3572 (0.0015) C13_$2 - 3.6100 (0.0013) C14_$2 - 3.7830 (0.0014) C15_$2Rms deviation of fitted atoms = 0.0005- 7.4963 (0.0028) *x* + 1.7481 (0.0065) *y* + 10.6592 (0.0082) *z* = 4.5261 (0.0067)Angle to previous plane (with approximate e.s.d.) = 30.79 (0.06)* 0.0118 (0.0008) C4 * -0.0134 (0.0008) C5 * 0.0021 (0.0009) C6 * 0.0111 (0.0009) C7 * -0.0128 (0.0009) C8 * 0.0013 (0.0009) C9Rms deviation of fitted atoms = 0.01015.6034 (0.0038) *x* + 5.0416 (0.0058) *y* + 12.9434 (0.0071) *z* = 8.2491 (0.0021)Angle to previous plane (with approximate e.s.d.) = 86.71 (0.03)* 0.0006 (0.0008) C10 * -0.0066 (0.0008) C11 * 0.0067 (0.0009) C12 * -0.0008 (0.0009) C13 * -0.0053 (0.0009) C14 * 0.0053 (0.0008) C15Rms deviation of fitted atoms = 0.004

### Fractional atomic coordinates and isotropic or equivalent isotropic displacement parameters (Å^2^)

**Table:**

	*x*	*y*	*z*	*U*_iso_*/*U*_eq_	
C1	0.12006 (12)	−0.00788 (8)	0.54798 (6)	0.0209 (3)	
C2	0.10916 (12)	0.08101 (8)	0.49535 (6)	0.0206 (3)	
C3	−0.01449 (12)	0.08355 (9)	0.44911 (6)	0.0215 (3)	
H3	−0.0224	0.1398	0.4156	0.026*	
C4	0.29282 (12)	−0.06850 (9)	0.64289 (6)	0.0219 (3)	
C5	0.27035 (13)	−0.17924 (10)	0.64288 (6)	0.0256 (3)	
H5	0.2148	−0.2119	0.6065	0.031*	
C6	0.32978 (14)	−0.24128 (10)	0.69630 (7)	0.0301 (3)	
H6	0.3123	−0.3164	0.6968	0.036*	
C7	0.41411 (15)	−0.19588 (10)	0.74902 (6)	0.0321 (3)	
H7	0.4531	−0.2392	0.7856	0.039*	
C8	0.44080 (15)	−0.08662 (10)	0.74762 (6)	0.0314 (3)	
H8	0.5013	−0.0551	0.7825	0.038*	
C9	0.37968 (13)	−0.02321 (9)	0.69556 (6)	0.0264 (3)	
H9	0.3969	0.0519	0.6956	0.032*	
C10	0.21200 (12)	0.24083 (9)	0.45179 (6)	0.0223 (3)	
C11	0.11033 (13)	0.32190 (10)	0.46367 (6)	0.0262 (3)	
H11	0.0366	0.3124	0.4982	0.031*	
C12	0.11658 (14)	0.41614 (9)	0.42529 (7)	0.0280 (3)	
H12	0.0483	0.4717	0.4343	0.034*	
C13	0.22200 (14)	0.43007 (10)	0.37364 (7)	0.0288 (3)	
H13	0.2254	0.4945	0.3469	0.035*	
C14	0.32205 (14)	0.34921 (10)	0.36147 (7)	0.0291 (3)	
H14	0.3940	0.3582	0.3260	0.035*	
C15	0.31853 (13)	0.25534 (9)	0.40038 (6)	0.0254 (3)	
H15	0.3887	0.2008	0.3921	0.031*	
H1	0.2849 (18)	0.0610 (13)	0.5831 (8)	0.030 (4)*	
N1	0.23876 (11)	0.00087 (8)	0.59090 (5)	0.0248 (2)	
N2	0.21592 (10)	0.14848 (8)	0.49516 (5)	0.0236 (2)	

### Atomic displacement parameters (Å^2^)

**Table:**

	*U*^11^	*U*^22^	*U*^33^	*U*^12^	*U*^13^	*U*^23^
C1	0.0178 (5)	0.0226 (5)	0.0223 (5)	0.0024 (4)	−0.0001 (4)	−0.0009 (4)
C2	0.0181 (5)	0.0207 (5)	0.0231 (5)	0.0007 (4)	0.0008 (4)	−0.0010 (4)
C3	0.0197 (5)	0.0220 (5)	0.0229 (5)	0.0005 (4)	−0.0010 (4)	0.0020 (4)
C4	0.0160 (5)	0.0264 (6)	0.0234 (5)	0.0013 (4)	0.0005 (4)	0.0022 (4)
C5	0.0198 (6)	0.0265 (6)	0.0305 (6)	−0.0003 (4)	−0.0015 (4)	−0.0008 (4)
C6	0.0243 (6)	0.0266 (6)	0.0393 (7)	0.0012 (5)	0.0003 (5)	0.0055 (5)
C7	0.0288 (7)	0.0374 (7)	0.0302 (6)	0.0062 (5)	−0.0025 (5)	0.0084 (5)
C8	0.0267 (7)	0.0395 (7)	0.0279 (6)	0.0026 (5)	−0.0061 (5)	−0.0014 (5)
C9	0.0233 (6)	0.0267 (6)	0.0291 (6)	0.0007 (4)	−0.0032 (4)	−0.0007 (4)
C10	0.0183 (5)	0.0222 (5)	0.0264 (6)	−0.0041 (4)	−0.0045 (4)	0.0007 (4)
C11	0.0201 (6)	0.0287 (6)	0.0297 (6)	−0.0013 (4)	0.0015 (4)	0.0006 (4)
C12	0.0225 (6)	0.0250 (6)	0.0364 (7)	0.0025 (4)	−0.0017 (5)	−0.0001 (5)
C13	0.0282 (6)	0.0251 (6)	0.0330 (6)	−0.0032 (5)	−0.0028 (5)	0.0064 (5)
C14	0.0244 (6)	0.0326 (6)	0.0301 (6)	−0.0035 (5)	0.0032 (5)	0.0038 (5)
C15	0.0202 (6)	0.0264 (6)	0.0296 (6)	0.0006 (4)	−0.0014 (4)	−0.0002 (4)
N1	0.0217 (5)	0.0237 (5)	0.0290 (5)	−0.0051 (4)	−0.0059 (4)	0.0044 (4)
N2	0.0194 (5)	0.0227 (5)	0.0286 (5)	−0.0017 (4)	−0.0019 (4)	0.0021 (4)

### Geometric parameters (Å, º)

**Table:**

C1—C3^i^	1.3547 (16)	C8—C9	1.3847 (17)
C1—N1	1.3662 (15)	C8—H8	0.9500
C1—C2	1.4957 (15)	C9—H9	0.9500
C2—N2	1.2927 (14)	C10—C15	1.3942 (17)
C2—C3	1.4365 (15)	C10—C11	1.3952 (17)
C3—C1^i^	1.3547 (16)	C10—N2	1.4160 (14)
C3—H3	0.9500	C11—C12	1.3840 (17)
C4—C5	1.3964 (16)	C11—H11	0.9500
C4—C9	1.3985 (16)	C12—C13	1.3893 (18)
C4—N1	1.4032 (15)	C12—H12	0.9500
C5—C6	1.3874 (17)	C13—C14	1.3842 (18)
C5—H5	0.9500	C13—H13	0.9500
C6—C7	1.3866 (19)	C14—C15	1.3846 (16)
C6—H6	0.9500	C14—H14	0.9500
C7—C8	1.3848 (19)	C15—H15	0.9500
C7—H7	0.9500	N1—H1	0.874 (17)
			
C3^i^—C1—N1	127.12 (10)	C8—C9—H9	119.6
C3^i^—C1—C2	119.71 (10)	C4—C9—H9	119.6
N1—C1—C2	113.12 (10)	C15—C10—C11	119.31 (10)
N2—C2—C3	125.78 (10)	C15—C10—N2	119.63 (10)
N2—C2—C1	115.69 (10)	C11—C10—N2	120.83 (10)
C3—C2—C1	118.51 (10)	C12—C11—C10	120.17 (11)
C1^i^—C3—C2	121.78 (10)	C12—C11—H11	119.9
C1^i^—C3—H3	119.1	C10—C11—H11	119.9
C2—C3—H3	119.1	C11—C12—C13	120.44 (11)
C5—C4—C9	118.94 (10)	C11—C12—H12	119.8
C5—C4—N1	123.87 (10)	C13—C12—H12	119.8
C9—C4—N1	117.12 (10)	C14—C13—C12	119.34 (11)
C6—C5—C4	119.55 (11)	C14—C13—H13	120.3
C6—C5—H5	120.2	C12—C13—H13	120.3
C4—C5—H5	120.2	C13—C14—C15	120.76 (11)
C7—C6—C5	121.32 (11)	C13—C14—H14	119.6
C7—C6—H6	119.3	C15—C14—H14	119.6
C5—C6—H6	119.3	C14—C15—C10	119.96 (11)
C8—C7—C6	119.14 (11)	C14—C15—H15	120.0
C8—C7—H7	120.4	C10—C15—H15	120.0
C6—C7—H7	120.4	C1—N1—C4	130.77 (10)
C9—C8—C7	120.25 (12)	C1—N1—H1	110.7 (10)
C9—C8—H8	119.9	C4—N1—H1	118.5 (10)
C7—C8—H8	119.9	C2—N2—C10	120.77 (10)
C8—C9—C4	120.74 (11)		
			
C3^i^—C1—C2—N2	−178.63 (10)	N2—C10—C11—C12	173.81 (11)
N1—C1—C2—N2	3.91 (14)	C10—C11—C12—C13	1.35 (18)
C3^i^—C1—C2—C3	0.14 (17)	C11—C12—C13—C14	−0.79 (19)
N1—C1—C2—C3	−177.31 (10)	C12—C13—C14—C15	−0.37 (19)
N2—C2—C3—C1^i^	178.50 (11)	C13—C14—C15—C10	0.95 (18)
C1—C2—C3—C1^i^	−0.14 (17)	C11—C10—C15—C14	−0.38 (17)
C9—C4—C5—C6	2.39 (17)	N2—C10—C15—C14	−175.02 (10)
N1—C4—C5—C6	179.27 (11)	C3^i^—C1—N1—C4	7.7 (2)
C4—C5—C6—C7	−1.51 (18)	C2—C1—N1—C4	−175.09 (11)
C5—C6—C7—C8	−0.8 (2)	C5—C4—N1—C1	26.84 (19)
C6—C7—C8—C9	2.2 (2)	C9—C4—N1—C1	−156.22 (12)
C7—C8—C9—C4	−1.33 (19)	C3—C2—N2—C10	6.60 (17)
C5—C4—C9—C8	−1.00 (18)	C1—C2—N2—C10	−174.73 (9)
N1—C4—C9—C8	−178.09 (11)	C15—C10—N2—C2	−119.41 (12)
C15—C10—C11—C12	−0.76 (17)	C11—C10—N2—C2	66.04 (14)

Symmetry code: (i) −*x*, −*y*, −*z*+1.
